# Supplementary material for: Study on the Metabolic Transformation Rule of Enrofloxacin Combined with Tilmicosin in Laying Hens
Source: Metabolites. 2023 Apr 6;13(4):528. doi: 10.3390/metabo13040528 (PMC10144589; doi:10.3390/metabo13040528)
Supplement: Supplementary file 1 [file metabolites-13-00528-s001.zip › metabolites-2256670-supplementary.pdf]

2.5 Method validation

2.5.1 Specificity of method

In this study, blank chicken egg homogenate samples were added with mixed solution (EF, CIP and TIM). Take blank chicken and egg homogenate sample and add appropriate amount of standard solution to make the required compound concentration in sample above the minimum quantitative limit, then the samples were extracted, purified and tested according to the above pretreatment method. In addition, take blank homogenate sample without adding standard sample and carry out the same operation. The peak times of EF, CIP and TIM were determined by comparing the chromatograms of blank and standard addition samples without interference from impurities was used as the criterion to determine the specificity of the method.

2.5.2 Limits of detection and limits of quantification

A series of concentrations of mixed standard solutions were added to blank egg homogenate samples, each sample was repeated 5 times. Extraction and purification were carried out according to "2.5.3" sample pretreatment method, and LC-MS/MS was determined. Each concentration was repeated for 3 times, and the operation was repeated for 5 times. The measured signal strength (S) and background signal strength (N) were recorded each time, and the mean value S and N of the measured results were taken for 25 times. The detection limitis defined by the concentration of the object to be measured when the Signal Noise Ratio (SNR) is S/N⩾3; limit of quantification (LOQ) is defined by the concentration of the substance to be measured when the SNR S/N⩾10[28].

2.5.3 The matrix matches the standard curve

Because matrix effect can affect sample detection, the method of matching standard working fluid with blank matrix was adopted in this study. Blank egg homogenate samples were extracted and purified by pre-treatment method, and a series of mixed standard solutions (EF, CIP and TIM) were added to prepare matrix standard solutions with concentrations of 10, 20, 50, 100, 200 and 500 μg/kg. Then the blank extract was diluted at an appropriate ratio, filtered by 0.22 μm filtration membrane, and detected by LC-MS/MS. With the concentration of the corresponding matrix standard

solution as the horizontal coordinate and the response area of the quantitative ion peak as the vertical coordinate, the matrix standard curve was drawn, the regression equation of the standard curve and the correlation coefficient were calculated, and the established method was evaluated on this basis.

2.5.4 Accuracy and precision

Accuracy refers to how close the measured value is to the real value or reference value. In this study, the recovery rate was used to represent accuracy; precision refers to the degree of variation repeatedly measured in the same sample, usually expressed as relative standard deviation (RSD), and in this study as the coefficient of variation between days and days. The suitable concentration of the standard solution (EF, CIP and TIM) was added into the blank homogenate sample of egg, and the concentrations of EF, CIP and TIM in the egg sample were 10, 50, 100 μg/kg (low, medium, high), respectively, and then LC-MS/MS was used for detection. According to the measured peak area of quantitative ions, the measured concentration is calculated by taking into the matrix standard curve obtained in the test. The recovery rate was calculated by the ratio of the measured concentration to the corresponding added concentration. Five replicates were set for each concentration, and the mean value was taken to calculate the coefficient of variation within days. Each concentration was repeated for 3 days and the coefficient of variation was calculated.
